# Patients with stable coronary artery disease and type 2 diabetes but without prior myocardial infarction or stroke and THEMIS-like patients: real-world prevalence and risk of major outcomes from the SNDS French nationwide claims database

**DOI:** 10.1186/s12933-021-01416-1

**Published:** 2021-11-25

**Authors:** Patrick Blin, Patrice Darmon, Patrick Henry, Estelle Guiard, Marie-Agnès Bernard, Caroline Dureau-Pournin, Hélène Maizi, Florence Thomas-Delecourt, Régis Lassalle, Cécile Droz-Perroteau, Nicholas Moore

**Affiliations:** 1 Univ. Bordeaux, INSERM CIC-P 1401, Bordeaux PharmacoEpi, 33000 Bordeaux, France; 2 Hospital La Conception, Marseille, France; 3 Aix-Marseille University, INSERM, INRA, C2VN, Marseille, France; 4 Hospital Lariboisiere, Paris, France; 5 AstraZeneca, Courbevoie, France

**Keywords:** Coronary artery disease, Type 2 diabetes mellitus, Prevalence, Myocardial infarction, Stroke, Mortality, Claims database

## Abstract

**Aim and hypotheses:**

The THEMIS randomized trial compared ticagrelor plus aspirin versus placebo plus aspirin for patients with stable coronary artery disease and type 2 diabetes mellitus (CAD-T2DM), and without prior myocardial infarction (MI) or stroke. The aim of the study was to quantify the size of the CAD-T2DM population without prior MI or stroke population in a real-world setting, and more specifically populations with similar THEMIS selection criteria (THEMIS-like and THEMIS-PCI-like populations), as well as their risk of major outcomes in current practice.

**Methods:**

A 2-year follow-up cohort study included all CAD-T2DM without MI/stroke prevalent patients on January 1st, 2014 in the SNDS French nationwide claims database. The THEMIS-like population concerned those ≥ 50 years of age with similar THEMIS inclusion and exclusion criteria. Prevalence was standardized to the European population. The cumulative incidence function was used to estimate the incidence of clinical outcomes (MI, ischemic stroke, and major bleeding according to the TIMI classification) with death as competing risk, and the Kaplan–Meier estimate for all-cause death and a composite outcome of MI, stroke and all-cause death.

**Results:**

From a population of about 50 million adults, the prevalence of CAD-T2DM without MI/stroke, THEMIS-like and THEMIS-PCI-like populations was respectively at 6.04, 1.50 and 0.27 per 1000 adults, with a mean age of 72.7, 72.3 and 70.9 years and less comorbidities and diabetic complications for the THEMIS-like and THEMIS-PCI-like population. The 2-year cumulative incidence was respectively 1.7%, 1.3% and 1.6% for MI, 1.7%, 1.5% and 1.4% for stroke, 4.8%, 3.1% and 2.9% for major bleeding, 13.6%, 9.7% and 6.8% for all-cause death, and 16.2%, 12.0% and 9.5% for the composite outcome.

**Conclusion:**

THEMIS-like prevalence was estimated at 1.50 per 1,000 adults, representing about a quarter of CAD-T2DM without MI/stroke patients, and 0.27 per 1000 adults for the THEMIS-PCI-like populations. In current French practice, the median age of both these populations was about 5–6 years older than in the THEMIS trial, with a 2-year incidence of major outcomes between two or four time above the ones of the placebo arm of the THEMIS trial using very close definitions. Registration No. EUPAS27402 (http://www.ENCEPP.eu).

## Introduction

Coronary artery disease (CAD) is one of the leading causes of mortality and morbidity, affecting almost 49 million people in the European Union, with a cost estimated at around €210 billion a year [1, 2]. Type 2 diabetes mellitus (T2DM) is a well-known risk factor for CAD, myocardial infarction (MI) and/or stroke [3–9]. Global estimates of T2DM in adults predict an increase from 415 million persons with T2DM (8.8%) in 2015 to 642 million (10.4%) in 2045, confirming the global impact of T2DM, especially in developing countries and impose a large economic burden on health care systems across the world [10]. In France, prevalence of treated T2DM was estimated at 2.6% in 2000 increasing to 4.4% in 2009 and 5% in 2015, using the nationwide claims database [11, 12]. Patients with both CAD and T2DM, and without MI or stroke history, are at high risk for cardiovascular events [13, 14].

In the THEMIS randomized clinical trial (RCT), ticagrelor plus aspirin was compared to a placebo plus aspirin for the prevention of cardiovascular death, MI or stroke in CAD-T2DM patients ≥ 50 years old without history of MI or stroke, receiving anti-hyperglycaemic drugs for at least 6 months and history of percutaneous coronary intervention (PCI) or coronary artery bypass graft (CABG) or angiographic evidence of ≥ 50% lumen stenosis of at least one coronary artery [15]. The incidence of ischemic cardiovascular events was lower in the ticagrelor group compared to placebo (7.7% versus 8.5%, p = 0.04) at 3 years of follow-up, whereas the incidence of major bleeding as defined by the TIMI (Thrombolysis in Myocardial Infarction) criteria was higher (2.2% versus 1.0%, p < 0.001). Furthermore, in the subpopulation of patients with prior PCI, ticagrelor plus aspirin also reduced the 3-year risk of ischemic events (7.3% versus 8.6%, p = 0.013), which was not the case for patients without prior PCI (p = 0.76), with still more major bleedings in both groups (2.0% versus 1.1%, p < 0.0001 and 2.4% versus 1.0%, p < 0.0001, respectively) [16].

The burden of the corresponding disease is not well known in the overall population. The aim of this study is to estimate the prevalence of the CAD-T2DM population without prior MI or stroke, and more specifically, of a THEMIS-like population, their characteristics and the risk of major outcomes in current practice, which might not be the same as in a highly selected RCT population [4, 17].

## Methods

### Design

The study design was a cohort including all CAD-T2DM prevalent patients on January 1st, 2014 (index date) from the main scheme of the SNDS French nationwide claims database with a 5-year history (2008–2013) and a follow-up of 2 years (2014–2015).

### Setting

The SNDS links national mandatory public health insurance system claims database to the national hospital-discharge summaries database system and the national death registry, using a unique national pseudonymised identifier [18]. It currently includes 98.8% of the French population, more than 66 million persons from birth (or immigration) to death (or emigration), even if a subject changes occupation or retires, and irrespective of socioeconomic status. The main scheme covers 86% of the French population and is the only one with a history before 2010 used to perform this study [18]. SNDS contains all reimbursed outpatient healthcare expenditures, public and private hospital-discharge summaries with International Classification of Diseases 10th revision (ICD-10) discharge diagnoses, and long-term disease (LTD) registration allowing 100% reimbursement for expenditure related to the LTD. Date of death is available and causes of death are uploaded gradually but were not available at the time of the study.

### Study population

Three populations were defined and analyzed: CAD-T2DM without MI-Stroke, THEMIS-like and THEMIS-PCI-like populations. CAD was defined as patients with at least one hospitalization within the 5-year history or LTD registration with ICD-10 codes I20-25 or coronary artery bypass graft (CABG) for CAD [18], ICD-10 I2I-23 for MI [19–21] and ICD-10 codes I63-I64 for ischemic or undefined stroke [22, 23]. T2DM was defined as patients with ≥ 3 non-insulin anti-diabetic dispensing or only long-acting insulin or a large packaging for a 3-month treatment within the year before index date or at least one hospitalization with ICD-10 codes E11 primary or secondary diagnosis between 2013 and 2014 or LTD registration [24]. THEMIS-like population was defined as CAD-T2DM adult patients ≥ 50 years without 5-year history of MI or stroke, or renal failure requiring dialysis, cirrhosis or liver cancer, intracranial bleeding, or gastro-intestinal bleeding for the last 6 months, or anticoagulant or P2Y12 inhibitor antiplatelet agent 2 months before or after index date. THEMIS-PCI-like populations as THEMIS-like population with a PCI within the 5-year history but no CABG.

### Outcomes

Outcomes were defined as a hospitalization with a primary diagnosis of the corresponding event. The main outcomes were MI (I21-I23 ICD-10 code), ischemic or unknown stroke (I63-I64 ICD-10 codes), all-cause death, and a composite of MI, stroke (I60-64 ICD-10 codes) and all-cause death, a composite cardiovascular (CV) event. Secondary outcomes were heart failure [25], and major bleeding defined by intracranial bleeding, or other bleeding with transfusion during hospital stay or fatal bleeding [22, 26], as a proxy for TIMI major bleeding classification.

### Statistical analysis

Prevalence was estimated for the three study populations and according to sex and three age-classes (18–64, 65–75, > 75 years) in France, and then standardized according to sex and 5-year age-classes for the European population, according to statistics from Eurostat, and the French population according to national statistics from INSEE. The cumulative incidence function was used to estimate primary and secondary clinical outcomes (i.e., MI, ischemic stroke, major bleeding, major organ specific bleeding and heart failure) with death as competing risk, and the Kaplan–Meier estimate for all-cause death and a composite of MI, stroke and all-cause death (composite outcome), for both study populations and for three age-classes (< 65, 65–75, > 75 years). Cox proportional hazards model, adjusted on gender, age, PCI and CABG history, was used to assess composite outcome risk factors among major comorbidities before index date (heart failure, atrial fibrillation, cerebrovascular disease, peripheral arterial disease, hypertension, dyslipidemia, chronic renal disease, renal impairment, chronic obstructive pulmonary disease, cancer), 56 SNDS pathology indicators, antidiabetic and CV drug exposure during the 2-year follow-up period as time-dependent covariates (considering that a treatment covers a 30-day period at each dispensing). Statistical analysis was conducted by Bordeaux PharmacoEpi using SAS^®^ software (SAS Institute, latest current version, North Carolina, USA).

## Results

**Fig. 1:**
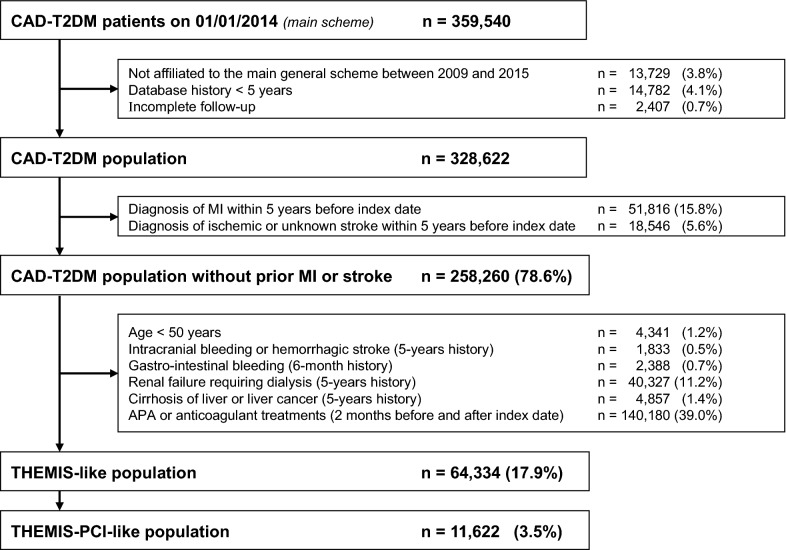
Patient flow-chart

On January 1st, 2014, 359,540 CAD-T2DM patients were identified within the main scheme of the SNDS database of whom 328,622 were continuously covered by the main scheme for all the study period with complete database history and follow-up. After excluding those with prior MI or stroke, 78.6%, 19.6% and 3.5% were included in the CAD-T2DM without prior MI or stroke, THEMIS-like and THEMIS-PCI-like population, respectively (Fig. 1).

The general characteristics of CAD-T2DM without MI/stroke and THEMIS-like population are similar with around two thirds of males, a mean 72 years, 55% with T2DM history of at least 5 years and 47% with CAD history of at least 5 years, including 26% with both T2DM and CAD history of at least 5 years, but comorbidity and diabetic complications were less frequent for the THEMIS-like population, as well as most of main care within the year before index date (Tables 1, 2). Compared to the THEMIS-like population, those with PCI were slightly younger with more men and no large difference for disease history, comorbidities, diabetic complications and main care within the year before index date, except for PCI history and three less frequent obstructive sleep apnea syndrome.

**Table 1:** Patient characteristics at index date for three study populations

	CAD-T2DM without MI/stroke population	THEMIS-like population	PCI THEMIS-like population
	n = 258,260	n = 64,334	n = 11,622
Age in years, mean (± SD)	72.7 (10.6)	72.3 (10.2)	70.9 (9.5)
Gender male, n (%)	176,407 (68.3)	42,238 (65.7)	8693 (74.8)
Disease history, n (%)			
T2DM Long Term Disease registration	199,425 (77.2)	50,060 (77.8)	9058 (77.9)
CAD Long Term Disease registration	111,419 (43.1)	28,144 (43.7)	6327 (54.4)
T2DM history ≥ 5 years	142,562 (55.2)	35,432 (55.1)	6386 (54.9)
CAD history ≥ 5 years	121,639 (47.1)	29,562 (46.0)	5264 (45.3)
Both CAD and T2DM history ≥ 5 years	67,040 (26.0)	16,346 (25.4)	3039 (26.1)
PCI within the 5-year history	71,576 (27.7)	11 657 (18.1)	11,622 (100)
CABG within the 5-year history	1860 (0.72)	335 (0.5)	0
Major comorbidities, n (%)			
Hypertension	204,943 (79.4)	48,554 (75.5)	8954 (77.0)
Atrial fibrillation	55,155 (21.4)	6647 (10.3)	992 (8.5)
Renal impairment	51,647 (20.0)	2943 (4.6)	429 (3.7)
Peripheral arterial disease	49,703 (19.2)	7326 (11.4)	1462 (12.6)
Dyslipidemia	49,406 (19.1)	10,673 (16.6)	2564 (22.1)
Cancer	47,066 (18.2)	10,958 (17.0)	1788 (15.4)
Heart failure	41,744 (16.2)	5821 (9.0)	932 (8.0)
Chronic obstructive pulmonary disease	35,558 (13.8)	7137 (11.1)	1092 (9.4)
Obstructive sleep apnea syndrome, n (%)	58,497 (22.7)	13,090 (20.3)	822 (7.1)
Diabetic complications, n (%)	101,419 (39.3)	20,301 (31.6)	3908 (33.6)
Diabetic foot ulcer	68,149 (26.4)	13,506 (21.0)	3077 (26,5)
Diabetic nephropathy	33,126 (12.8)	4299 (6.7)	645 (5.5)
Diabetic retinopathy	27,715 (10.7)	5400 (8.4)	776 (6.7)
Diabetic neuropathy	25,891 (10.0)	4874 (7.6)	667 (5.7)

SD, standard deviation; T2DM, type 2 diabetes mellitus; CAD, coronary artery disease, PCI, Percutaneous coronary intervention; CABG, coronary artery bypass graft

**Table 2:** Main care within the year before index date for the three populations

	CAD-T2DM without MI/stroke population	THEMIS-like population	PCI THEMIS-like population
	n = 258,260	n = 64,334	n = 11,622
General practitioner visit number, mean (± SD)	10.2 (7.1)	9.5 (6.8)	9.5 (6.6)
Cardiologist visit number, mean (± SD)	2.3 (2.8)	2.0 (2.3)	2.1 (2.4)
Other specialist visit number, mean (± SD)	5.3 (7.4)	4.7 (6.0)	4.6 (6.0)
At least one			
HbA1c test, n (%)	22,1237 (85.7)	52,830 (82.1)	9666 (83.2)
Hospitalization, n (%)	141,149 (54.7)	30,215 (47.0)	4963 (42.7)
PCI, n (%)	24,413 (9.5)	1429 (2.2)	1328 (11.4)
Antidiabetic drug, n (%)	227,959 (88.3)	54,769 (85.1)	10,037 (86.4)
CV drug, n (%)	244,660 (94.7)	58,550 (91.0)	10,697 (92.0)
ASA, n (%)	164,045 (63.5)	45,829 (71.2)	8566 (73.7)
P2Y12i antiplatelet agent, n (%)	102,843 (39.8)	7372 (11.5)	1703 (14.7)
Anticoagulant treatment, n (%)	62,371 (24.2)	6273 (9.8)	1182 (10.2)
Other CV drugs, n (%)	2429,64 (94.1)	58,034 (90.2)	10,598 (91.2)
Statins	200,790 (77.7)	45,935 (71.4)	8445 (72.7)
ACEI or ARB	199,125 (77.1)	46,335 (72.0)	8467 (72.9)
Beta blockers	172,165 (66.7)	38,290 (59.5)	7157 (61.6)
Diuretics	108,141 (41.9)	22,106 (34.4)	4087 (35.2)
Calcium channel blockers	94,237 (36.5)	21,280 (33.1)	3907 (33.6)
Organic nitrates	55,079 (21.3)	10,552 (16.4)	2043 (17.6)
Other lipid modifying agents than statins	34,418 (13.3)	7900 (12.3)	1484 (12.8)
Other vasodilators used in cardiac diseases	27,003 (10.5)	5431 (8.4)	1029 (8.9)

SD: Standard Deviation; T2DM: type 2 diabetes Mellitus; CAD: Coronary artery disease; PCI: Percutaneous Coronary Intervention; CABG: coronary artery bypass graft

The prevalence rate for the European population was 6.04 per 1000 persons for CAD-T2DM without MI/stroke population, 1.50 for the THEMIS-like population and 0.27 for the THEMIS-PCI-like population, with an estimation of about 2.5 million, 620,000 and 110,000 patients in Europe, respectively (Table 3). The prevalence rate for the French population was very close corresponding to about 317,000, 79,000 and 14,000 patients in France, respectively. The prevalence was higher for men than women in both populations and increased with age. For all patients, as well as according to gender and age classes, the THEMIS-like population represented a quarter of all CAD-T2DM without prior MI or stroke.

**Table 3:** Prevalence the three study populations on January 2014 for the European and French adult populations

		CAD-T2DM without MI/stroke population		THEMIS-like population		PCI THEMIS-like population	
		Prevalence^a^ (n)^b^		Prevalence^a^ (n)^b^		Prevalence^a^ (n)^b^	
European adults (≥ 18 years)^c^							
Total	**411,670,644**	6.04	2,484,478	1.50	617,215	**0.27**	**(112,644)**
Male	**198,490,922**	8.74	1,735,311	2.09	415,164	0.43	(85,559)
Female	**213,179,722**	3.51	749,167	0.95	202,051	0.13	(27,085)
18–64 years	**317,653,393**	1.73	58,870	0.46	145,927	**0.09**	**(29,145)**
Male	**158,513,473**	2.71	44,884	0.70	110,216	0.15	(24,334)
Female	**159,139,920**	0.76	13,986	0.22	35,711	0.03	(4811)
65–75 years	**53,161,006**	16.88	897,267	4.37	232,529	**0.87**	**(46,137)**
Male	**24,570,561**	27.74	681,573	6.95	170,793	1.49	(36,557)
Female	**28,590,445**	7.54	215,694	2.16	61,736	0.34	(9580)
> 75 years	**40,856,245**	25.38	1,037,002	5.84	238,759	**0.91**	**(37,362)**
Male	**15,406,888**	40.54	624,650	8.71	134,155	1.60	(24,668)
Female	**25,449,357**	16.20	412,352	4.11	104,604	0.50	(12,794)
French adults (≥ 18 years)^d^							
Total	**51,341,304**	**6.17**	(316,824)	**1.53**	(78,597)	**0.28**	**(14,169)**
Male	24,468,453	8.96	(219,319)	2.14	(52,371)	0.44	(10,723)
Female	26,872,851	3.63	(97,505)	0.98	(26,226)	0.13	(3446)
18–64 years	**39,478,257**	**1.78**	(70,169)	**0.47**	(18,681)	**0.09**	**(3731)**
Male	19,448,237	2.81	(54,633)	0.72	(14,082)	0.16	(3112)
Female	20,030,020	0.78	(15,536)	0.23	(4599)	0.03	(619)
65–75 years	**6,341,554**	**16.73**	(106,122)	**4.34**	(27,552)	**0.86**	**(5475)**
Male	2,964,622	27.40	(81,218)	6.88	(20,397)	1.47	(4368)
Female	3,376,932	7.37	(24,904)	2.12	(7155)	0.33	(107)
> 75 years	**5,521,493**	**25.45**	(140,533)	**5.86**	(32,364)	**0.90**	**(4963)**
Male	2,055,594	40.61	(83,468)	8.70	(17,892)	1.58	(3243)
Female	3,465,899	16.46	(57,065)	4.18	(14,472)	0.50	(1720)

Bold value indicates the size of the cooresponding EU population, i.e. 411.670644 Millions adults in EU

T2DM, type 2 diabetes mellitus; CAD, coronary artery disease

^a^rate for 1000 adults

^b^n = estimated number of patients per 1000 adults

^c^Standardized from Eurostat Population

^d^Standardized from INSEE Population

The 2-year incidence of primary and secondary outcomes was lower in the THEMIS-like population than for all CAD-T2DM without MI/stroke, but higher than for the THEMIS-PCI-like population (Table 4). For the composite outcome (MI, stroke or all-cause death), it was 16.2% for CAD-T2DM without MI/stroke, 12.0% for the THEMIS-like and 9.5% for the THEMIS-PCI-like with death as the most common event, 13.6%, 9.7% and 6.8, respectively, and similar and relatively low incidence for MI (1.7%, 1.3% and 1,6%, respectively) and ischemic stroke (1.7, 1.5% and 1.4%, respectively) (Table 4). While heart failure is more frequent (9.5%, 5.3% and 4.2%, respectively). For major bleeding, the 2-year incidence was 4.8%, 3.1% and 2.9%, respectively, including approximatively a quarter of fatal bleeding for the three population (1.2%, 0.7% and 0.6%, respectively). There was a clear age-related increase in all event incidences, especially marked for heart failure and all-cause death, and except for MI which increased very little for those above 75 years old (Table 4).

**Table 4:** Outcome cumulative incidence (%) at 2-year follow-up for all patients and according to age for the three study populations

	CAD-T2DM without prior MI or stroke				THEMIS-like population				PCI THEMIS-like population			
	All	< 65 years	[65–75] years	> 75 years	All	< 65 years	[65–75] years	> 75 years	All	< 65 years	[65–75] years	> 75 years
Composite outcome (MI, stroke & all-cause death)	**16.2**	**7.5**	**11.4**	**24.7**	**12.0**	**5.2**	**7.8**	**20.0**	**9.5**	**5.9**	**7.0**	**15.2**
Myocardial infarction	1.7	1.6	1.6	1.8	1.3	1.3	1.2	1.5	1.6	1.8	1.2	1.9
Ischemic stroke	1.7	0.9	1.4	2.4	1.5	0.8	1.2	2.3	1.4	1.0	1.1	2.0
All-cause death	13.6	5.2	8.9	21.9	9.7	3.3	5.7	17.3	6.8	3.3	4.8	12.0
Heart failure	**9.5**	**4.3**	**7.1**	**14.2**	**5.3**	**2.3**	**3.6**	**8.7**	**4.2**	**2.0**	**3.1**	**7.2**
Major bleeding	**4.8**	**2.9**	**4.3**	**6.3**	**3.1**	**1.6**	**2.5**	**4.5**	**2.9**	**1.5**	**2.8**	**4.2**
Intracranial bleeding	0.6	0.3	0.5	0.8	0.4	0.2	0.3	0.7	0.4	0.1	0.4	0.5
Other bleeding with transfusion	4.1	2.5	3.7	5.2	2.5	1.3	2.0	3.6	2.5	1.3	2.4	3.5
Fatal bleeding	1.2	0.5	1.0	1.8	0.7	0.2	0.6	1.3	0.6	0.2	0.6	0.9

Bold value indicates correspond to main criteria and none blod value to subgoup of the main criteria, i.e. the composite = 16.2 and subcriteria = 1.7, 1.7, 13.6 and some subjet may have 2 events : an MI and died later

T2DM, type 2 diabetes mellitus; CAD, coronary artery disease; PCI, percutaneous coronary intervention

In the three populations, the occurrence of the composite outcome (MI, stroke or all-cause death) was associated with male sex, increasing with age, previous history of heart failure, cancer, liver disease, peripheral arterial disease, or mood disorder (Table 5). Patients of the CAD-T2DM without MI/stroke and the THEMIS-like population with PCI history presented a lower risk for the composite outcome. During the follow-up, the event rate increased with the use of diuretics and insulin compared with the antidiabetic monotherapy probably from an association with the severity of heart disease and diabetes, respectively (Table 5). The use of antiplatelet agent did not modify the event rates for CAD-T2DM without prior MI or stroke patients, but was associated with a higher risk in the THEMIS-like population, and was probably more a proxy of disease severity as for diuretics and insulin above (Table 5).

**Table 5:** Factors Associated with the composite outcome in the three study populations multivariate Cox proportional hazards risk model, selection threshold HR ≥ 1.20 or HR ≤ 0.80, adjusted on PCI and CABG 5-year history)

	CAD-T2DM population without prior MI or stroke n = 258,260			THEMIS-like population n = 64,334			PCI THEMIS-like population n = 11,622		
	No	Yes	HR [95% CI]	No	Yes	HR [95% CI]	No	Yes	HR [95% CI]
Male^a^	148,849	27,558	1.25 [1.22–1.27]	37,396	4842	1.31 [1.25–1.38]	7902	791	1.14 [0.99–1.31]
Age (in years)									
< 55	11,412	712	1.00	2334	102	1.00	460	22	1.00
55–59	15,739	1210	1.18 [1.08–1.29]	4757	225	1.04 [0.82–1.31]	908	45	0.98 [0.59–1.63]
60–64	27,295	2502	1.37 [1.26–1.49]	7794	483	1.34 [1.08–1.66]	1561	117	1.42 [0.90–2.23]
65–69	37,571	4244	1.64 [1.51–1.77]	10,421	758	1.53 [1.24–1.88]	2089	133	1.19 [0.76–1.87]
70–74	34,402	4804	1.95 [1.80–2.11]	9143	857	1.92 [1.56–2.36]	1818	151	1.52 [0.97–2.37]
75–79	35,768	6698	2.49 [2.30–2.69]	8946	1164	2.55 [2.09–3.13]	1677	198	2.00 [1.29–3.12]
80–84	31,106	8954	3.51 [3.25–3.79]	7438	1536	3.78 [3.09–4.62]	1267	204	2.54 [1.63–3.95]
85–89	16,767	7687	5.07 [4.69–5.48]	4066	1453	5.98 [4.88–7.31]	577	165	4.13 [2.64–6.47]
≥ 90	6373	5016	7.74 [7.15–8.38]	1713	1144	9.88 [8.06–12.1]	166	64	5.10 [3.12–8.32]
PCI within the 5-year history	62,072	9504	0.88 [0.86–0.90]	10,555	1102	0.86 [0.81–0.92]		11,622	–
CABG within the 5-year history	1544	316	0.95 [0.85–1.06]	304	31	0.73 [0.51–1.04]		0	–
Within the 1-year history									
Heart failure^b^	15,240	7783	1.77 [1.73–1.82]	2278	892	1.79 [1.67–1.92]	316	105	2.05 [1.67–2.52]
Cancer^b^	26,547	9521	1.67 [1.63–1.71]	6777	1728	1.70 [1.61–1.79]	1166	249	1.84 [1.59–2.12]
Liver diseases^b, c^	5176	1882	1.80 [1.72–1.89]	580	82	1.16 [0.93–1.44]	78	14	1.64 [0.96–2.78]
Peripheral arterial disease^b^	20,657	6996	1.56 [1.52–1.61]	2957	799	1.72 [1.59–1.85]	561	149	2.21 [1.85–2.63]
Neurotic and mood disorders^b^	11,996	3377	1.29 [1.25–1.34]	3221	660	1.33 [1.23–1.45]	393	66	1.47 [1.14–1.89]
Within the 2-year follow-up									
Diuretics^d^			1.49 [1.46–1.52]			1.44 [1.37–1.51]			1.62 [1.42–1.85]
Antiplatelet agent^d^			1.03 [1.01–1.06]			1.29 [1.13–1.46]			1.24 [0.96–1.60]
Antidiabetic treatment^d^									
Monotherapy			1.00			1.00			1.00
No antidiabetic treatment			0.72 [0.69–0.75]			0.72 [0.65–0.80]			0.82 [0.60–1.11]
Bitherapy			0.69 [0.65–0.72]			0.70 [0.63–0.79]			0.80 [0.57–1.13]
Tritherapy or more			0.65 [0.60–0.70]			0.67 [0.56–0.79]			0.73 [0.45–1.17]
Insulin			1.27 [1.21–1.32]			1.20 [1.08–1.34]			1.41 [1.03–1.93]

PCI, percutaneous coronary intervention; CABG, coronary artery bypass graft

^a^Reference is female

^b^Reference is non-presence of variable

^c^Excluding chronic viral hepatitis/cystic fibrosis

^d^Time dependent variables during follow-up

## Discussion

The prevalence of the CAD-T2DM without prior MI or stroke, THEMIS-like populations and THEMIS-PCI-like populations, estimated with sex-age standardization for the European adult population, was 6.04, 1.50 and 0.27 per 1000 persons, respectively, increasing with age and with a higher prevalence for men. Thus, the THEMIS-like population represented a quarter of all CAD-T2DM without prior MI or stroke, as well as for men, women and each age-class. The 2-year cumulative incidence of main outcome was higher for all CAD-T2DM without MI/stroke than for the THEMIS-like population, which was itself higher than the THEMIS-PCI-like population except for MI (1.7, 1.3% and 1.6%): 1.7%, 1.5% and 1.4% for stroke, 4.8%, 3.1% and 2.9% for major bleeding, 13.6%, 9.7% and 6.8% for all-cause death, 16.2%, 12.0% and 9.5% for the composite of MI, stroke and all-cause death, respectively.

The patients included in the THEMIS-like and THEMIS-PCI-like population approximate those included in the THEMIS RCT, with similar inclusion criteria but not exactly the same. However, it was close to criteria that would be applied for therapeutic indication in current practice after such trial. Our results showed that the THEMIS-like population have same mean age, sex-ratio, T2DM and CAD history duration than all CAD-T2DM without prior MI or stroke patients, but a lower rate of comorbidities and diabetic complications, than can be explained by THEMIS exclusion criteria, especially renal failure requiring dialysis and liver cancer or cirrhosis, and probably also anticoagulant treatment or antiplatelet agent prescribed for other comorbidities.

Compared to the patients in the THEMIS RCT placebo arms [15], our THEMIS-like populations was about 6-year older (72.3 vs. 66.0 years) with same sex-ratio and duration of diabetes, a little more peripheral arterial disease (11.4% vs. 9.0%) and diabetes complications (31.6% vs. 25.3%), but less coronary arterial revascularization 18.6% within the 5-year history versus 79.9% any time with a median of 4.1 years for the most recent. The rate of hypertension and dyslipidemia was also lower (75.5% vs. 92.4% and 16.6% vs. 87.1%, respectively), but probably related to poor recording, which contrasts with 71.4% use of statins, and 72.0% use of ACE inhibitors or angiotensin receptor blockers, 33.1% use of calcium channel blockers within the year before index date. One patient out of 6 (17.0%) had cancer, but this was not specified for THEMIS trial. Similar differences were observed for the THEMIS-PCI-like population [16].

In the THEMIS RCT, outcome incidences were also estimated using the Kaplan–Meier estimate but reported after 3 years of follow-up. We estimate the cumulative incidence at 2-years as two-thirds of the 3-year result, considering constant increase during time according to the figures of the publications [15, 16]. The 2-year cumulative incidence of the composite of MI, any stroke, or all-cause death was 12.0% for the THEMIS-like population, the double than for the same outcome of the placebo arm of the THEMIS RCT (first Exploratory outcomes). The difference is related to all-cause death with a three times higher 2-year cumulative incidence (9.7% vs. 3.2%), whereas this incidence was closer for ischemic stroke (1.5% vs. 1.2%) and lower for MI (1.3% vs. 2.2%). Similar differences were observed for the THEMIS-PCI-like population with about 5 years difference (70.9 vs. 66.0 years) [16]. The main outcome of THEMIS RCT unfortunately, could not be estimated in our study because of the unavailability of cardiovascular mortality. However, the non-cardiovascular death added only 20% more events between the primary outcome in the placebo group (7.6%) and the explonary outcome “Death from any cause, myocardial infarction, or stroke” (9.2%)”. The 6-year difference of age between the two studies could explained a part of the death difference, but a significant proportion should correspond to patient selection in RCT, as described by P.G. Steg with the same ratio of 1 to 3 [17]. For major bleeding, the 2-year incidence is also much higher, about 4 times higher, 3.1% compared to 0.7% at 2 years of follow-up (1.0 at 3 years).

The major bleeding TIMI classification includes intracranial bleeding, fatal bleeding, and hemorrhage associated with a drop in hemoglobin of ≥ 5 g/dL or a ≥ 15%. Since no lab test result was available in the SNDS database, for this last criterion we used the proxy of hospitalization with bleeding main diagnosis and transfusion during hospital stay, considering that such drop of hemoglobin is an indication for a transfusion and few transfusions should be prescribed for a bleeding with a lower hemoglobin decrease. The large difference of bleeding could be partially linked to other drugs used in real-life setting. If we excluded patients who took P2Y12 inhibitor antiplatelet agents or anticoagulants 2 months before or after index date, 11.5% of included patients used P2Y12 inhibitor antiplatelet before, and then several patients started P2Y12 inhibitor antiplatelet agent or anticoagulant during the follow-up, while patients treatment was not authorized in the THEMIS RCT. Whatever, we had found similar distortion between patients included in the PEGASUS-TIMI 54 trial and a similar real-life population, in which higher all-cause bleeding rates and death were also found in real-life than in the clinical trial [4].

The SNDS is a national healthcare claims database linked to the national hospital discharge summaries database that covers about 99% of the French population. It provides a unique opportunity to identify all CAD-T2DM patients, with exhaustive information about reimbursed treatments out of hospital and use of reimbursed healthcare resources, as well as all hospitalizations. Furthermore, patient records from an existing database are not impacted by the study. The SNDS has already been used to study the type of patients in this study, including for secondary prevention post MI [15, 20], or to describe long-term patient outcomes after MI [5].

The main limitation of this claims and hospitalization database is that it was built for administrative and reimbursement purposes with little clinical data and no biological results, including severity or stage of the disease or some risk factors such as diet, environmental exposure, obesity, alcohol, family history, smoking status, and no information about drug adherence, though the full history of drug dispensing may be consistent with adherence, even though medicines that are prescribed are not always taken. This is common in all studies of drug utilization and effects, even with direct access to the patients. The results of lab tests can often be inferred by subsequent medical prescriptions or tests (e.g., measure of transaminases followed by hospital admission for hepatic injury).

Since all patients identified are extracted from a national database, there is no study selection bias, nor attrition bias; patients are included in the database from birth or immigration to death or emigration, irrespective of employer, income, age or social status [18]. Since deaths are recorded in the database using the national death registry, there is no information bias for this outcome. Causes of death were not available at the time of the study in the SNDS, and the primary outcome of THEMIS RCT cannot be estimated in our study, but we used the THEMIS exploratory outcome of MI, any stroke and death for any cause for comparison between both studies. The ICD-10 coding of hospital discharge summaries is regularly audited by the national health insurance system and there is cross-validation of coding between hospitals. In addition, a number of diagnostic codes and strategies such as for MI, heart failure or stroke have been verified with positive predictive values over 90%, improving over time [27]. The same outcomes have been used in previous studies in the same database [4, 20, 22, 26]. All information in the database is fully independent from the study and there is no reason that any potential miscoding will be different between drugs or populations. Some T2DM patients could be misclassified as type 1 because of insulin therapy. To prevent this classification bias, a 5-year history period was used to investigate prior treatment sequences with sufficient delay to select appropriate T2DM patients.

## Conclusion

The THEMIS-like population European prevalence was estimated at 1.50 per 1000 adults, representing about a quarter of CAD-T2DM without MI/stroke patients, in current French practice, with similar general characteristics and less comorbidities, and 0.27 per 1000 adults for the THEMIS-PCI-like populations. The median age of the THEMIS-like and THEMIS-PCI-like populations was about 5–6 years older than in the THEMIS trial, and the 2-year incidence of major outcomes was well above compared to the placebo arm of the THEMIS trial using very close definitions, about double for the composite outcome, triple for deaths and quadruple for major bleedings than those of the placebo arm of THEMIS RCT. Such lower risk of patients in clinical trials than in real-life is a common observation, and justifies regular verification of drug performances in real-life and the external validity of clinical trials to real-life use of the medications.

## Data Availability

According to the French law, it is forbidden to share individual data from a SNDS data extraction. However, any researcher from a European entity can submit a file to the Health Data Hub (https://www.health-data-hub.fr, new name for the National Institute of Health Data cited above) to have access to the same SNDS data extraction.

## Abbreviations

CAD-T2DM: Coronary artery disease
CABG: Coronary artery bypass graft
CV: Cardio vascular
ICD-10: International classification of diseases 10th revision
INSEE: Institut National de la Statistique et des Etudes Economiques
LTD: Long-term disease
MI: Myocardial infarction
PCI: Percutaneous coronary intervention
RCT: Randomized clinical trial
SNDS: Système National des Données de Santé
T2DM: Type 2 diabetes mellitus
TIMI: Thrombolysis in myocardial infarction
